# Genetic Variation and Cardiovascular Risk Factors: A Cohort Study on Migrants from the Former Soviet Union and a Native German Population

**DOI:** 10.3390/ijerph18126215

**Published:** 2021-06-08

**Authors:** Marianne Huebner, Daniela Börnigen, Andreas Deckert, Rolf Holle, Christa Meisinger, Martina Müller-Nurasyid, Annette Peters, Wolfgang Rathmann, Heiko Becher

**Affiliations:** 1 Institute for Biometry and Epidemiology, University Medical Center Hamburg-Eppendorf, Martinistraße 52, 20246 Hamburg, Germany; huebner@msu.edu; 2 Department of Statistics and Probability, Michigan State University, East Lansing, MI 48864, USA; 3 Bioinformatics Core Facility, University Medical Center Hamburg-Eppendorf, Martinistraße 52, 20246 Hamburg, Germany; d.boernigen@uke.de; 4 Institute of Global Health, Epidemiology and Biostatistics, University Hospital Heidelberg, Im Neuenheimer Feld 324, 69120 Heidelberg, Germany; a.deckert@uni-heidelberg.de; 5 Institute of Health Economics and Health Care Management, Helmholtz Zentrum München, GmbH, 85764 Neuherberg, Germany; rholle@ibe.med.uni-muenchen.de; 6 German Research Center for Environmental Health, Institute of Epidemiology, Helmholtz Zentrum München, 85764 Neuherberg, Germany; c.meisinger@unika-t.de (C.M.); peters@helmholtz-muenchen.de (A.P.); 7 Institute of Genetic Epidemiology, Helmholtz Zentrum München—German Research Center for Environmental Health, 85764 Neuherberg, Germany; martina.mueller@helmholtz-muenchen.de; 8 German Center for Diabetes Research (DZD), München-Neuherberg, 85764 Neuherberg, Germany; wolfgang.rathmann@ddz.de; 9 German Diabetes Center, Institute for Biometrics and Epidemiology, 40225 Duesseldorf, Germany

**Keywords:** migrants, resettlers, genetic differences, cardiovascular diseases, GWAS, lifestyle, Germany

## Abstract

Resettlers are a large migrant group of more than 2 million people in Germany who migrated mainly from the former Soviet Union to Germany after 1989. We sought to compare the distribution of the major risk factors for cardiovascular disease (CVD) and to investigate the overall genetic differences in a study population which consisted of resettlers and native (autochthone) Germans. This was a joint analysis of two cohort studies which were performed in the region of Augsburg, Bavaria, Germany, with 3363 native Germans and 363 resettlers. Data from questionnaires and physical examinations were used to compare the risk factors for cardiovascular diseases between the resettlers and native Germans. A population-based genome-wide association analysis was performed in order to identify the genetic differences between the two groups. The distribution of the major risk factors for CVD differed between the two groups. The resettlers lead a less active lifestyle. While female resettlers smoked less than their German counterparts, the men showed similar smoking behavior. SNPs from three genes (BTNL2, DGKB, TGFBR3) indicated a difference in the two populations. In other studies, these genes have been shown to be associated with CVD, rheumatoid arthritis and osteoporosis, respectively.

## 1. Introduction

Germany is a country of immigration, and according to recent data about 25% of the German population has a migrant background. One large migrant group are the so-called Aussiedler (resettlers) from Eastern European countries, with a large subgroup immigrating from the former Soviet Union (FSU) to Germany. The ancestors of the resettlers emigrated to the Russian empire in the 18th and 19th century, by invitation of the government. They were privileged compared to the Russian population, but at the beginning of the 20th century they became victims of persecution and suffered increasing discrimination. Many ethnic Germans were deported to Kazakhstan and Siberia in 1941. After the opening of the inner-German border in 1989, the majority of the resettlers from the former Soviet Union migrated to Germany. The highest number of migrants came to Germany between the years 1990 and 1995 [1,2].

There are about 3.2 Million resettlers living in Germany, which is approximately 3.5% of the total German population. Although the resettlers were quasi-randomly assigned to different regions in Germany, they are overrepresented in some regions. In the region of Augsburg, a city in Bavaria with about 300,000 inhabitants, the proportion of resettlers is about six percent (https://statistikinteraktiv.augsburg.de/, accessed on 6 March 2019).

In the FSU, the overall mortality—and in particular CVD mortality—was much higher in recent decades compared to that in Germany and other Western countries [3,4]. Due to the expectation that there could also be a high mortality among resettlers, register-based cohort studies on resettlers were performed [2]. However, these studies showed a significantly lower all-cause and cardiovascular disease mortality compared to the German population. 

Recent data show that about 90% of the population in the former FSU with German ancestors migrated to Germany, of which very few migrated back after some time in Germany. To date, about 400,000 people who consider themselves to be ethnic Germans continue to live in Russia according to the 2010 census of the Russian Federation [5]. Therefore, common explanations for a low mortality in migrants, such as the healthy migrant effect and salmon bias (i.e., immigrant groups appear to be healthier than they are because less healthy individuals selectively return to their country of origin), are unlikely to explain the findings.

A few studies on risk factor prevalence for the migrant population [6,7] showed that the typical risk factors for CVD are high among resettlers. Thus, these observations do not explain the observed pattern of low CVD mortality. Because the history of this migrant group suggests that they are the descendants of a group of Germans who not only decided to migrate from their home country in the 18th century but also survived difficult living conditions, there may be differences in the genetic predisposition conveying a survival advantage. Genetic stratification may exist within racial/ethnic groups. In a population-based genome-wide association analysis between five European populations, several genes were reported to be stratified within European populations [8]. A genetic cluster analysis in a US study with whites, African Americans, East Asians and Hispanics of 326 microsatellite markers produced four major clusters, which showed near-perfect correspondence with the four self-reported race/ethnicity categories [9]. It is unknown whether there is such a clustering for the resettlers and the native (autochthone) German population. Phenotypes controlled by a dozen or fewer loci can be expected to show substantial overlap between human populations [10].

This is a joint analysis of two prospective cohort studies which were sampled from the same total population. One is the so-called KORA S4 cohort, which was recruited in the years 1999–2001 as an age- and sex-stratified sample [7,11]. The other is a cohort of resettlers [12]. Both cohorts are described in more detail below. In this study, we investigated whether the observed differences in CVD mortality from previous large register-based studies can partly be explained by a differing distribution of genetic factors that contribute to this disease group. In addition, we analysed the distribution of other risk factors for CVD. The underlying hypothesis is that the ancestors of the resettlers may have been selected because they were particularly healthy and physically advantaged, and therefore may have been genetically advantaged compared to the average population. Our objectives were to compare the distribution of major risk factors for cardiovascular diseases and to investigate the overall genetic differences in a study population which consists of resettlers and native Germans from the region of Augsburg. We sought to examine the magnitude of the differences in the SNP allele frequencies between the two populations. 

## 2. Materials and Methods

### 2.1. Study Population 

The study sample originates from two cohort studies which were both performed in the region of Augsburg, Bavaria, Germany.

### 2.2. KORA Cohort 

**Figure 1:**
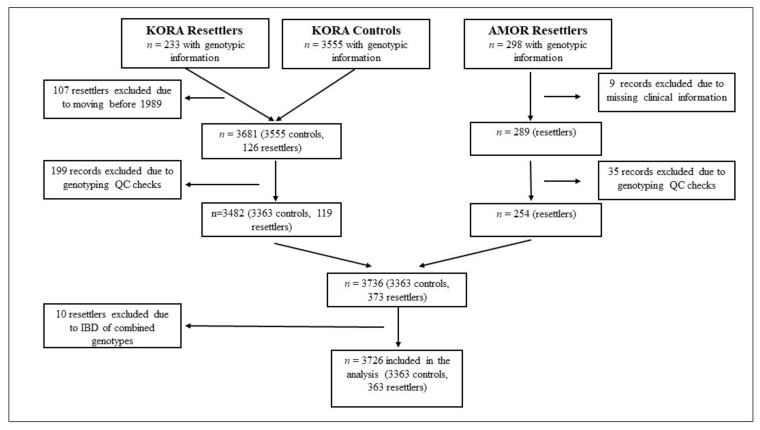
STROBE flow chart.

The KORA study is a series of population-based surveys conducted in the city of Augsburg in Southern Germany and its two adjacent counties. The cohort KORA S4 was recruited in the years 1999–2001 as an age- and sex-stratified sample of German residents based on information from local registry offices. The study participants underwent intensive clinical examinations and answered health questionnaires. Overall, the cohort consisted of 3788 individuals. It included 233 resettlers (Figure 1).

After excluding resettlers who moved before 1989 and excluding records due to genotyping QC checks, the final sub-cohort contained 3363 native Germans and 119 resettlers. Genotyping was performed on the Affymetrix Axiom. The details of the cohort are given in [7,11].

### 2.3. AMOR Cohort 

Resettlers living in the greater Augsburg region in 2010 (*n* = 3718) were identified in the population registry. They were asked by written letter to provide a self-administered questionnaire (based on the KORA study questionnaire). The respondents then were invited to take part in a detailed physical examination (anthropometric measures and blood pressure measurements) and a personal interview, and to provide a blood sample. The data collection took place from 2011 to 2013. From the cohort of 673 individuals who provided questionnaire data, 298 provided blood samples for genetic testing. Of these, both the clinical examination and interview data were available for 180 subjects (63 males, 117 females). The genotyping was performed on the Illumina Infinium Global Screening Array GSA v.1.0 (Illumina Inc., San Diego, CA, USA). A comparison between the Illumina and Affymetrix platforms (Thermo Fisher Scientific, Waltham, MA, USA) indicated very high agreement [13]. More details of the AMOR cohort are given in [12].

Both cohorts combined thus included 531 resettlers (233 from KORA and 298 from the AMOR cohorts) and 3555 individuals as a comparison group (the KORA native German population) with genotypic information and questionnaire data. Of these, clinical examination data were available from 413 resettlers (233 from KORA and 180 from AMOR). People who participated in both studies could be detected through genetic identity, and were excluded from the analysis.

### 2.4. Data Preprocessing 

For the genetics data, we implemented quality control measures, namely the QC pipeline by the eMERGE network [14]. The SNPs were excluded if: (i) the SNP genotyping call rate was < 0.99; (ii) the minor allele frequency (MAF) was < 0.01; (iii) the linkage disequilibrium (LD) was < 0.2; and (iv) the Hardy–Weinberg Equilibrium (HWE) *p* < 10 ×10^−6^. At the sample level, the (v) heterozygosity cutoff was 0.1 and (vi) the kinship coefficient was < 0.1. A principle component analysis (PCA) was performed in order to detect the outliers.

Variables from the questionnaires used in the KORA and AMOR cohorts were harmonized across the surveys. The hypertension categories were determined from the systolic and diastolic blood pressure, measured according to the National Joint Committee [15]. The measurement units were standardized for the serum measurements. The estimated glomular filtration rate (eGFR) was calculated using the CKD-EPI equation [16].

### 2.5. Statistical Methods 

The continuous variables were summarized for the resettlers and native Germans with medians and quartiles, categorical variables as frequencies, and percentages. The self-reported comorbidity variables were myocardial infarction (MI), stroke, diabetes and cancer. The comorbidities derived from the laboratory measurements were hypertension and chronic kidney disease (CKD). The laboratory measurements were available for the KORA cohort and for a subset of the AMOR cohort, including systolic and diastolic blood pressure, triglycerides, creatinine, cholesterol, waist and hip measurements, height, and weight. The demographic variables and lifestyle factors included age, sex, sport activity, family status, education level, smoking status, and the family history of MI and stroke. These variables were described for the resettlers and native Germans, and were stratified by sex, as there are known sex differences. A conditional logistic regression analysis was used to analyze the differences in the risk factor distribution between resettlers and native Germans in a multivariable model. The strata were formed by 5-year age groups. 

In order to investigate a possible general genetic difference between the resettlers and Germans, we first performed a principal component analysis (PCA) based on all of the SNPs available for both cohorts. A genome-wide association analysis was conducted with ‘resettler’ as the outcome, adjusted for age and sex. The *p*-values were adjusted for the Bonferroni criterion, and the false discovery rate (FDR) threshold was set at 0.1. The GWAS catalogue [17] was searched in order to observe whether the resulting SNPs and genes were identified in an association analysis for cardiovascular diseases, diabetes, or cancer. The SNPs thus identified were entered into unadjusted and adjusted logistic regression models with cardiovascular disease (MI or stroke) or diabetes as the outcomes for the entire population. The SNPs were coded as additive with the homozygous genotype for the minor allele coded as 2, the heterozygous genotype coded as 1, and otherwise as 0. Due to the possibility that there is an overrepresentation of subjects with cardiovascular disease among the resettlers, the GWAS was repeated after removing such cases.

The statistical analyses were performed using R version 3.5.1 (R Foundation for Statistical Computing: Vienna, Austria), including the packages SNPRelate v.1.12.2 [18] and snpStats v 1.28.0 [19] from the Comprehensive R Archive Network [20]. This study was reported according the guidelines of the STROBE Statement [21] and the STREGA checklist [22].

## 3. Results

Both cohorts combined included 531 resettlers (233 from KORA and 298 from the AMOR cohort) and 3555 individuals as a comparison group (the KORA native German population). The flow diagram (Figure 1) describes the reasons for various exclusions. Ten resettlers participated in both studies and were detected through their genetic identity. The final study group for the analysis consisted of 3726 individuals (363 resettlers and 3363 in the comparison group) for which both the genetic data and the data from the interview and examination were available. After the preprocessing, 50,340 SNPs were in common for the KORA and AMOR SNP arrays. The age range for the resettlers was 20 to 86 years, and 24 to 75 years for the native Germans. The resettlers were on average 5 years older for both women and men. The distribution of the demographic factors, CVD risk factors, anthropometric measurements and laboratory measurements are described in Table 1.

The results of the univariable and the multivariable conditional logistic regression models with age in 5 year increments, in order to compare the resettlers and the native Germans stratified by gender, are shown in Table 2. The resettlers showed lower physical activity and a higher BMI. While the smoking prevalence in males was similar, with an OR for current male smokers of 1.02 (95% CI: 0.65, 1.61), *p* = 0.940 in comparison to native Germans, female resettlers smoke considerably less than the native German women, with an OR of 0.35 (95% CI: 0.22, 0.55), *p* < 0.001. Hypertension (normal, pre-hypertension, stage I and stage II) did not show a statistically significant different distribution in either gender. The cholesterol levels in the resettlers were lower for female resettlers (OR for women 0.94 (95% CI: 0.90, 0.98), *p* = 0.006; OR for men 0.97 (95% CI: 0.93, 1.02); *p* = 0.207), and the triglyceride levels were higher (OR for women 1.05 (95% CI: 1.03, 1.07), *p* < 0.001; OR for men 1.02 (95% CI: 1.00, 1.04); *p* = 0.021). Diabetes was more frequent in resettlers for both sexes (OR for women 2.82 (95% CI: 1.63, 4.86); *p* < 0.001; OR for men 2.34 (95% CI: 1.28, 4.23); *p* = 0.006).

**Figure 2:**
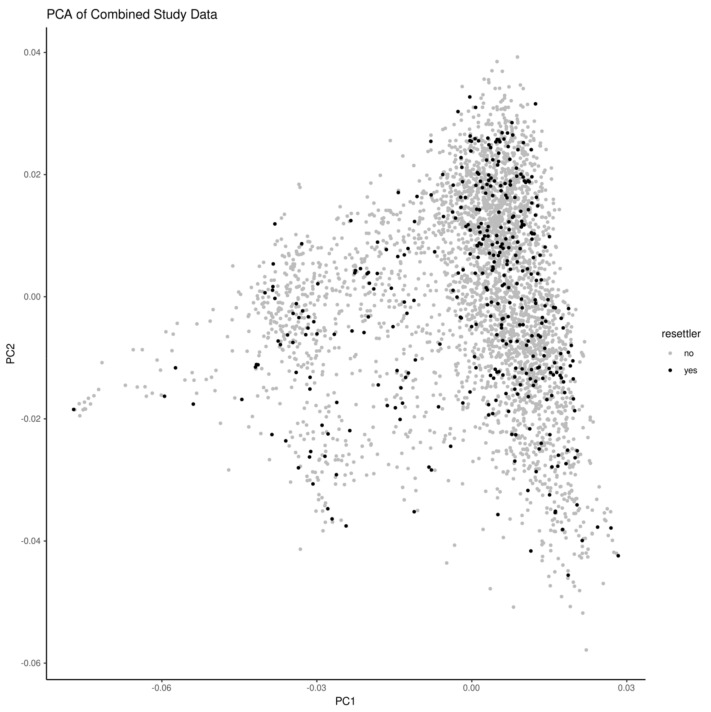
Principle component analysis (PCA) of the combined KORA and AMOR single nucleotide polymorphisms, with an indication of resettler status.

SNPs in the regions of 10 genes that have been shown to stratify European populations [8] were in the set of the 50K SNPs. However, none of these SNPs were shown to differentiate resettlers and native Germans in our dataset. A principal component analysis on all of the SNPs also did not indicate a separation between the resettlers and native Germans (Figure 2).

**Figure 3:**
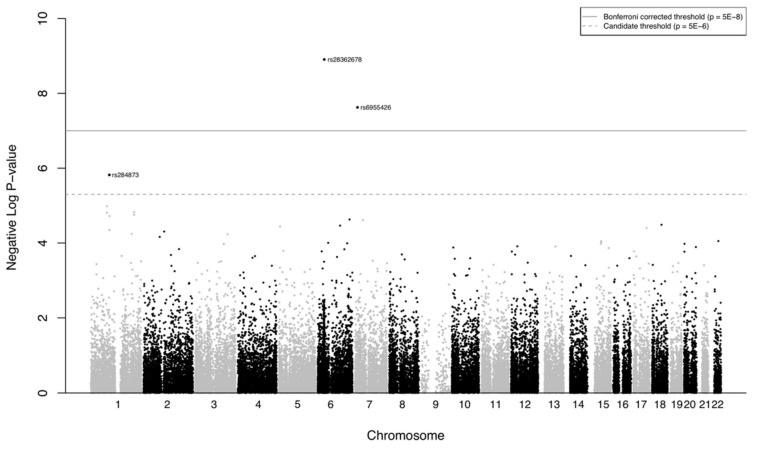
Manhattan plot for the genome-wide analysis of resettler status.

In a GWAS with resettler status as the outcome, three loci reached genome-wide significance to indicate a difference in the two populations after adjusting for sex and age, and correcting for multiple testing (Figure 3, Table 3). 

These three SNPs belong to the genes DGKB, BTNL2, and TGFBR3 that were indicated in previous genome wide association studies with CVD (DGKB), rheumatoid arthritis and sarcoidosis (BTNL2), and bone mass and osteoporosis (TGFBR3), respectively. 

In a sensitivity analysis, we performed a GWAS excluding self-reported cases of previous myocardial infarction (20 resettlers, 66 native Germans); the same SNPs from BTNL2 and TGFBR3 marked a genetic difference between the resettlers and native Germans. An additional SNP rs11579207 from TGFBR3 was also associated with resettler status.

## 4. Discussion

This is the first study in which resettlers were investigated regarding a general genetic difference to the native German populations and their genetic predisposition for cardiovascular diseases. In addition, the study contributed to the previously limited information on risk factors in this migrant group. Because previous studies showed a lower CVD mortality in resettlers and, contrary to this finding, a higher prevalence of CVD risk factors, we hypothesized that genetic factors may partly explain this observation.

### 4.1. Gender Differences of Cardiovascular Disease Risk Factors in Resettlers

In order to explain the observed differences in disease incidence or mortality between different populations, the first natural analysis is a comparison of the known medical or lifestyle risk factors. The previous studies were small; however, they pointed towards a higher prevalence of CVD risk factors in resettlers [6,7]. Several of the main risk factors for CVD showed a different distribution in resettlers; however, it must be noted that the proportion of missing information was higher in resettlers, and a clear missing at random assumption is not justified. In both men and women, BMI and physical activity had a less favorable distribution in resettlers, which are indicators for a potentially higher CVD mortality. In men, smoking and hypertension, two other major risk factors for CVD, showed a similar distribution in resettlers as in native Germans. In women, smoking is much less common in female resettlers compared to native German women, but normal blood pressure has a lower prevalence. Overall, the results are partly in line with the prior data and, in our view, would indicate a higher CVD risk in resettlers. 

### 4.2. Genetic Differences between Resettlers and Native Germans

The PCA analysis showed no general genetic difference between both groups; however, the GWAS analysis provided some interesting results. Genetic association studies with a large number of tests carry the risk of spurious results. In the present study, significance was retained at the genome-wide level after correction for multiple testing. Furthermore, we explored the functional relevance of the genes indicated in other association studies.

BTNL2 belongs to the butyrophilin-like B7 family of immunoregulators. Direct interaction between the BTN and its receptor on activated T cells leads to the suppression of the T cell response. TGFBR3 is a major mediator of TGF-beta signaling pathways and also functions as a BMP cell-surface receptor. Diacylglycerol kinases (DGKs) are regulators of the intracellular concentration of diacylglycerol and thus play a key role in cellular processes [24]. In genome-wide association studies, these genes have been identified in connection with myocardial infarction (DGKB [24]); immune function, rheumatoid arthritis, and sarcoidosis (BTNL2 [23]); and bone mass in different ethnic groups (TGFBR3 [25]). While these studies examined different SNPs of the same genes not included in our dataset, the SNP rs28362678 (BTNL2) identified in our study as having different variants for resettlers and native Germans has been shown to be associated with rheumatoid arthritis [23]. 

### 4.3. Limitations

There are several limitations. First, while the previous studies on the mortality of the resettlers were based on registries in which selection bias can be excluded, this study was based on the voluntary participation of individuals. The response rate was low, especially in the AMOR cohort. This may, in particular, have had an effect on the observed risk factor distribution and on self-reported disease. The resettler cohort consisted of older subjects than the native Germans, and this contributed to the comorbidity load. Second, due to our use of two different genotype platforms, the GWAS was limited to about 50K SNPs in common on both arrays. Third, the phenotypic data are missing for 22 percent of all of the resettlers because some individuals from the AMOR subcohort only provided a blood sample and did not undertake the physical examinations. This was because the blood samples were provided through the home physician, while all of the examinations were performed in the study center, which these individuals did not attend due to time constraints. We consider it unlikely, however, that the genetic information is correlated with participation, and thus we believe that our results are unbiased. Fourth, it was difficult to motivate the resettlers in the AMOR study to participate. The original study protocol assumed a much larger sample size. 

Another set of risk factors for CVD are environmental exposures, such as air pollution [26]. The resettlers originated from several states in the former Soviet Union, which would make an exposure assessment almost impossible. Therefore, it is difficult to make comparisons of the exposure levels to the Augsburg area. We do not think, however, that possible differences in air pollution levels could explain the lower CVD mortality in the resettlers.

Our cohorts were not selected for specific diseases, and it would be desirable to follow up our results with further studies such as the German National Cohort NAKO [27], which recently finished its recruitment phase. It includes a random sample from 18 study centers widely distributed in Germany, with a study size of 205,000 participants, of whom 3500 are resettlers. A detailed assessment of disease risk factors, an extensive medical examination program and a collection of various biomaterials was performed. The data cleaning processes are underway, and analyses will follow in the near future.

## 5. Conclusions

This is the first study in which resettlers were investigated regarding a general genetic difference to the native German population. The gene DGKB was shown to be associated with CVD, but the SNP rs6955426 is a novel locus on this gene and should be considered further in future studies. The minor allele is a risk allele, and resettlers have a higher minor allele frequency. The SNP rs28362678 on BTNL2 has previously been shown to be associated with rheumatoid arthritis, and gene function related to diabetes. TGFBR3 has been shown to be associated with osteoporosis. The distribution of the CVD risk factors, such as BMI or physical activity, is less favorable in resettlers compared to the native Germans. Further ongoing studies, in particular the German National Cohort (NAKO) which includes about 3500 resettlers, with detailed data on physical examinations, questionnaire data and biologic specimens, will provide more insights on the genetics and the mortality and morbidity pattern of this migrant group. 

## Figures and Tables

**Table 1:** Population characteristics of the resettlers and native Germans, stratified by sex.

	Women		Men	
	Resettlers (*n* = 214)	Native Germans (*n* = 1727)	Resettlers (*n* = 149)	Native Germans (*n* = 1636)
Age (years)	54 (44, 62)	49 (37,60)	55 (43, 66)	51 (37, 62)
missing	4	0	0	0
Family Status				
single	161 (9.3%)	19 (9.0%)	5 (3.4%)	213 (13.0%)
cohabit	145 (68.4%)	1243 (72.0%)	126 (84.6%)	1255 (76.8%)
separated	19 (9.0%)	177 (10.3%)	13 (8.7%)	130 (8.0%)
widowed	145 (8.4%)	29 (13.7%)	5 (3.4%)	36 (2.2%)
missing	1	1	0	2
Anthropometrics				
Height (cm)	159.5 (156.2, 163.6)	162 (157.7, 166.5)	172.5 (168.2, 177.5)	175.1 (170.3, 179.8)
missing	56 (26.2%)	7 (0.4%)	34 (22.8%)	4 (0.2%)
Weight (kg)	76 (66.1, 85.0)	68.3 (60.7, 77.6)	84.4 (74.7, 93.6)	82.7 (75.4, 91.0)
missing	56 (26.2%)	18 (1.0%)	34 (22.8%)	6 (0.4%)
BMI (kg.m^−2^)	29.8 (25.6, 33.4)	25.9 (22.9, 29.6)	28.3 (25.6, 30.9)	27.0 (24.9, 29.6)
missing	56 (26.2%)	19 (1.1%)	34 (22.8%)	6 (0.4%)
Waist-to-hip ratio	0.84 (0.78, 0.88)	0.80 (0.76, 0.85)	0.93 (0.89, 0.97)	0.95 (0.90, 1.0)
missing	56 (26.2%)	18 (1.0%)	34 (22.8%)	4 (0.2%)
Laboratory measurements				
Cholesterol (mg/dl)	219 (196,248)	222 (196, 255)	215 (191, 243)	224 (198, 256)
missing	56 (26.2%)	7 (0.4%)	35 (24.6%)	8 (0.5%)
Triglycerides (mg/dl)	149 (105, 223)	109 (83, 154)	161 (110, 265)	131 (91, 196)
missing	102 (47.7%)	1045 (60.5%)	72 (50.7%)	910 (55.6%)
Creatinine (mg/dl)	0.77 (0.70,0.87)	0.75 (0.68, 0.82)	0.98 (0.87, 1.10)	0.93 (0.84, 1.02)
missing	57 (26.6%)	18 (1.0%)	35 (24.6%)	19 (1.2%)
EGFR	86.8 (73.2, 100.7)	93.9 (81.3, 105.4)	88.0 (71.2, 102.2)	93.3 (83.0, 104.5)
missing	59 (27.6%)	18 (1.0%)	35 (24.6%)	19 (1.2%)
Lifestyle factors				
Smoking				
Never	174 (82.9%)	911 (52.8%)	48 (32.7%)	530 (32.5%)
Previous	13 (6.2%)	451 (26.1%)	60 (40.8%)	628 (38.5%)
Current	23 (11.0%)	364 (21.1%)	39 (26.5%)	475 (29.1%)
missing	3	1	2	3
Physical activity				
Regular	41 (20.1%)	872 (50.6%)	40 (28.2%)	816 (50.1%)
Irregular	50 (24.5%)	297 (17.2%)	23 (16.2%)	292 (17.9%)
Inactive	113 (55.4%)	555 (32.2%)	79 (55.6%)	522 (32.0%)
missing	10	3	7	6
Hypertension				
normal	61 (39.4%)	800 (46.4%)	19 (16.5%)	323 (19.8%)
pre	61 (39.4%)	558 (32.4%)	57 (49.6%)	708 (43.4%)
Stage 1	26 (16.8%)	284 (16.5%)	26 (22.6%)	418 (25.6%)
Stage 2	7 (4.5%)	81 (4.7%)	13 (11.3%)	181 (11.1%)
missing	58 (27.1%)	4 (0.2%)	34 (22.8%)	6 (0.4%)

The data are presented as the median (quartiles) or as *n* (%) for the categorical variables. Abbreviations: EGFR—estimated glomular filtration rate.

**Table 2:** Univariable and multivariable logistic regression analysis to identify the differences in the risk factors for cardiovascular diseases between resettlers and native Germans.

	Women *n* = 1941		Men *n* = 1785	
	OR * (95% CI)	OR ** (95% CI)	OR * (95% CI)	OR ** (95% CI)
Number of resettlers (total) in model		146 (1830)		107 (1713)
BMI	1.10 (1.07,1.14); *p* < 0.001	1.08 (1.05, 1.12); *p* < 0.001	1.04 (1.00,1.09); *p* = 0.051	1.02 (0.98, 1.08): *p* = 0.329
Cholesterol (10 mg/dl)	0.94 (0.90, 0.98); *p* = 0.006	0.94 (0.89, 0.99); *p* = 0.015	0.97 (0.93, 1.02); *p* = 0.207	0.98 (0.94, 1.03); *p* = 0.524
Triglycerides (10 mg/dl)	1.05 (1.03, 1.07); *p* < 0.001		1.02 (1.00,1.04); *p* = 0.021	
EGFR	0.98 (0.97, 0.99); *p* < 0.001	0.97 (0.96, 0.98); *p* < 0.001	0.98 (0.97, 0.99); *p* = 0.006	0.98 (0.97, 0.99); *p* = 0.007
Smoking				
never	1	1		1
previous	0.16 (0.09, 0.28); *p* < 0.001	0.22 (0.12, 0.41); *p* < 0.001	0.99 (0.66, 1.45); *p* = 0.949	1.12 (0.68, 2.05); *p* = 0.665
current	0.35 (0.22, 0.55); *p* < 0.001	0.37 (0.21, 0.65); *p* < 0.001	1.02 (0.65, 1.61); *p* = 0.940	1.19 (0.69,2.05); *p* = 0.537
Physical activity				
regular	1	1		1
irregular	3.87 (2.49, 6.03); *p* < 0.001	4.24 (2.48, 7.25); *p* < 0.001	1.63 (0.96, 2.78); *p* = 0.075	1.32 (0.70, 2.50); *p* = 0.388
inactive	4.26 (2.90, 6.28); *p* < 0.001	4.53 (2.79, 7.36); *p* < 0.001	2.92 (1.94, 4.40); *p* < 0.001	2.57 (1.62, 4.10); *p* < 0.001
Hypertension				
normal	1	1	1	1
Pre-hypertension	1.16 (0.78, 1.73); *p* = 0.456	0.83 (0.54, 1.29); *p* = 0.416	1.41 (0.81, 2.44); *p* = 0.226	1.32 (0.74, 2.35); *p* = 0.346
Stage 1	(0.97 (0.58, 1.62); *p* = 0.913	0.46 (0.25, 0.83); *p* = 0.011	1.01 (0.53, 1.92); *p* = 0.970	0.89 (0.45, 1.76); *p* = 0.736
Stage 2	0.95 (0.41, 2.23); *p* = 0.914	0.40 (0.15, 1.05); *p* = 0.062	1.07 (0.50, 23.0); *p* = 0.860	1.02 (0.45,2.30); *p* = 0.956

* Conditional logistic regression, with age in 5 yearage groups; ** conditional logistic regression, with age in 5 yearage groups; all of the risk factors were included in the model.

**Table 3:** SNPs differentiating resettlers and native Germans, and the distribution of the genotypes.

	rs28362678	rs6955426	rs284873
Chromosome: Gene	6: BTNL2	7: DGKB	1: TGFBR3
Gene function	Immunoregulators	Cellular processes	Cell surface receptor
Diseases *	Rheumatoid arthritis, sarcoidosis	Myocardial infarction	Bone mass and osteoporosis
Alleles	C > T	G > A	T > C
MAF (resettlers/controls)	5.8%/14.4%	19.5%/12.35%	13.46%/8.2%
AA	7(1.9%)/52 (1.5%)	8(2.2%)/39 (1.2%)	8 (2.2%)/20 (0.6%)
AB	28(7.7%)/864 (25.7%)	126 (34.6%) /750 (22.3%)	82 (22.5%)/510 (15.2%)
BB	326 (89.8%)/2443 (72.6%)	229 (63.2%)/2562 (76.2%)	273 (75.3%)/2829 (84.1%)
missing	2 (0.5%)/4 (0.1%)	0/12 (0.4%)	0/4 (0.1%)
*p*-value	1.24 × 10^−9^	2.37 × 10^−8^	1.51 × 10^−6^
FDR	6.26 × 10^−5^	0.000598	0.025374

Abbreviations: MAF—minor allele frequency; FDR—false discovery rate. ‘A’ denotes the minor allele and ‘B’ denotes the major allele. * BTNL2 [23]; DGKB [24]; TGFBR3 [25].

## Data Availability

The data are subject to national data protection laws; restrictions were imposed by the Ethics Committee of the Bavarian Medical Association to ensure the data privacy of the study participants and therefore the data cannot be made freely available in a public repository. The data are third party and belong to the KORA research platform, but can be accessed for specific research projects through individual project agreements. Interested researchers can request the data from KORA via the KORA.passt online tool (https://epi.helmholtz-muenchen.de/ accessed on 1 June 2021). In a data request, one has to briefly describe the intended scientific question and then select the variables of interest within the KORA.passt tool. We confirm that interested researchers who agree to the general terms and conditions of the KORA data user agreement can access the data of KORA in the same way we did. All other relevant data are within the manuscript and its Supporting Information files.
